# Corrigendum

**DOI:** 10.1002/jmrs.199

**Published:** 2016-12-02

Wu VWC, Lam Y‐N. Radiation‐induced temporo‐mandibular joint disorder in post‐radiotherapy nasopharyngeal carcinoma patients: assessment and treatment. *J Med Radiat Sci* 2016; **63**: 124–132. DOI: 10.1002/jmrs.145.

The authors would like to draw the reader's attention to an error in the following article:

The following statement should be included under Funding Information:

This study is funded by Hong Kong General Research Fund (GRF No:563412).

The authors apologise for this error and any confusion it may have caused.

